# The Relationship Between Technology Use and Colon Cancer Screening

**DOI:** 10.1093/geroni/igaa057.1014

**Published:** 2020-12-16

**Authors:** Na Sun

**Affiliations:** Miami University, Oxford, Ohio, United States

## Abstract

Population aging is accompanied by an increase in chronic diseases such as cancer. Colon cancer is the third most common cancer and a leading cause of cancer death. Screening tests can aid early detection and treatment. It is unclear how information and communication technology (ICT), especially media through mobile devices, influences cancer screening. This study analyzes the relationship between ICT usage and colon cancer screening among U.S. adults. Data are from the second cycle of the Health Information National Trend Survey 5. Cancer screening included having one of the following: colonoscopy, sigmoidoscopy and/or stool blood test to check for colon cancer. Approximately 70% of respondents had at least some college education, 51% were female, and the mean age was 48 years. More than half of respondents report using apps on a tablet or smartphone for health and wellness purposes, and around 70% of them used apps for health communication and decision-making. Based on results of a binary logistic regression model, people who use mobile apps for discussions with health care providers (p<0.01), who use the internet to look for information about cancer (p <0.01), and who do not use mobile apps to make decisions about how to treat an illness or condition (p<0.01) are more likely to conduct cancer screening. ICT usage may enable people to gather information about cancer screening and improve patient and physician communication. Future studies should explore longitudinal associations between ICT usage, cancer screening, and cancer outcomes.

